# Association between exposure to environmental pollutants and increased oral health risks, a comprehensive review

**DOI:** 10.3389/fpubh.2024.1482991

**Published:** 2025-01-06

**Authors:** Li Zhu, Mengchen Tang, Yinyin Cai, Panpan Wang

**Affiliations:** ^1^Guangdong Provincial Key Laboratory of Stomatology, Guanghua School of Stomatology, Sun Yat-sen University, Guangzhou, China; ^2^Institute of Atmospheric Environmental Economics, Nanjing University of Information Science and Technology, Nanjing, China; ^3^Shenzhen Institute of Meteorological Innovation, Shenzhen, China; ^4^Department of Periodontology, Guanghua School and Hospital of Stomatology, Sun Yat-sen University, Guangzhou, China

**Keywords:** oral diseases, environmental pollution, contaminant, air pollution, chemical pollution, heavy metals, secondhand smoke

## Abstract

The burden of disease and death attributable to environmental pollution is a growing public health challenge worldwide, particularly in developing countries. While the adverse effects of environmental pollution on oral health have garnered increasing attention, a comprehensive and systematic assessment remains lacking. This article delves into the intricate relationship between environmental pollution and oral health, highlighting significant impacts on various aspects such as dental caries, periodontal diseases, oral facial clefts, cancer, as well as other oral diseases. Our results suggested that secondhand smoke, particulate matters (PM) and heavy metals are the most important risk factors affecting oral health. Additional contributors, such as radiation pollutants, electronic cigarette, phthalates, gaseous air pollutants, pesticides, solvents, wood dust, formaldehyde and excessive fluoride were investigated, though evidence for their impacts remains limited and often inconclusive. The review also explores potential mechanisms underlying these impacts, including microorganism, inflammation, oxidative stress, genetic influences, and toxicant exposures from heavy metals and other pollutants. For instance, PM2.5 may contribute to dental caries by disrupting oral pH balance and absorbing heavy metals such as lead and cadmium which have been considered as caries promoting elements. It is also associated with adverse inflammatory responses and tissue damage in periodontal tissues by causing oxidative stress, potentially leading to periodontitis. Drawing on current evidence, it provides a comprehensive analysis of these associations, offering critical insights to guide the development of preventive strategies and public health interventions. The findings highlight the pressing need for future research to validate the causal links between environmental pollution and oral diseases and to unravel the underlying biological mechanisms. Ultimately, greater attention must be directed toward addressing the relationship between environmental pollution and oral diseases, with a focus on pollution control and the reduction of preventable environmental risks to safeguard oral health on a broader scale.

## Graphical abstract

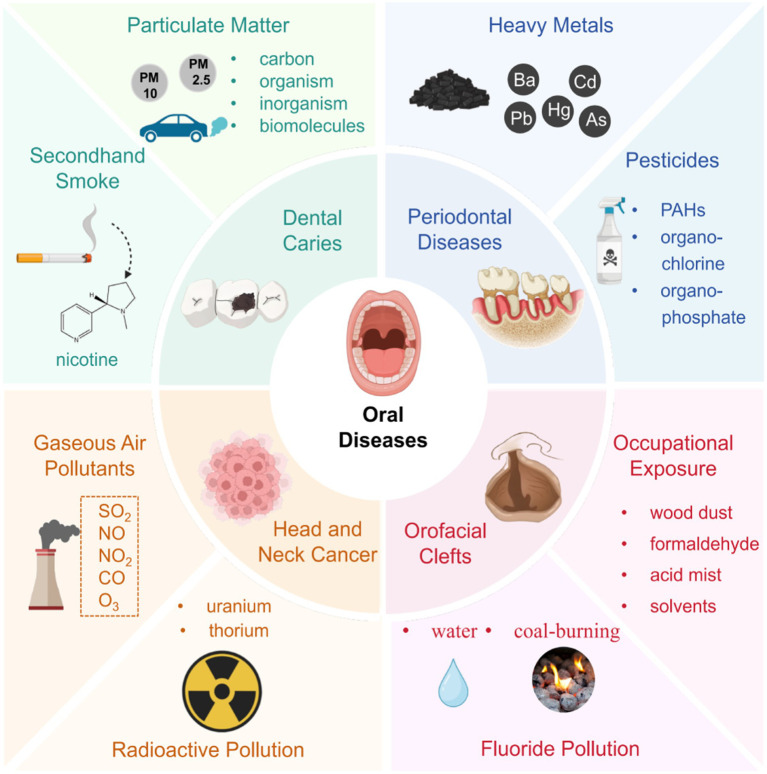

## 1 Introduction

Oral diseases encompass a spectrum of conditions affecting the oral tissues, impacting over 3.5 billion individuals globally as per the Global Burden of Disease Study 2015 (1). Among these, dental caries, periodontal disease, tooth loss, and oral cavity cancers are prominent, exerting substantial adverse effects on individual well-being, economic resources, and healthcare systems worldwide (2). Recent years have witnessed a growing acknowledgment of the critical role of oral health, with increasing attention to its nexus with general health (3, 4).

Environmental pollution, characterized by the contamination of air, water bodies, and land with hazardous substances, poses a significant threat to human health and the environment. The Global Burden of Disease Study 2019 reports approximately 9 million annual deaths attributable to environmental pollution, highlighting its pervasive impact and global health implications, particularly accentuated in developing nations (5). Modern forms of pollution, notably ambient air and chemical pollution are emerging as major contributors to this burden (6). Air pollution constitutes a complex mixture of gaseous components, solid and liquid particles, frequently exceeding standard concentrations. Air pollution is a significant threat to health in developed and developing countries (7, 8). Acknowledged repercussions of air pollution encompass respiratory ailments (9, 10), cardiovascular incidents (11), central nervous system dysfunctions (12), cancer (13, 14) and other health detriments (15). Most of the burden from air pollution is attributed to particulate matter (PM), which is variable particles with minuscule diameters (16). Concurrently, gaseous constituents such as nitrogen oxides, carbon monoxide, ozone, sulfur oxides, dioxins, and polycyclic aromatic hydrocarbons (PAHs) also contribute to air pollution’s composition (17). Recently, with the continuous increase of smokers globally, the health effects of indoor air pollutants such as secondhand smoke (SHS) have also received widespread attention. Additionally, environmental chemical pollution has increased substantially over the past 20 years on a global scale. Chemicals including heavy metals, pesticides, organic solvents et al. have become widely disseminated in the global environment and are proved to be harmful to human health.

Numerous epidemiological studies have hinted at a potential link between environmental pollution and the etiology of various oral diseases, including dental caries, periodontal diseases, orofacial clefts (OFCs), cancer and others. Investigations into exposure to environmental contaminants globally underscore the urgency of elucidating their role as risk factors for oral diseases. Therefore, a concerted effort is warranted to comprehensively elucidate this association. This review synthesizes epidemiological evidence concerning the correlation between oral diseases and environmental pollutants, encompassing SHS, PM, heavy metals and other environmental risk factors. Additionally, it seeks to elucidate potential mechanistic pathways underlying these associations. By delineating the sources of harmful chemicals in our environment and assessing their impact on oral health, this review endeavors to provide crucial insights for regulatory strategies and guide future research endeavors in the realm of oral disease causation.

## 2 Environmental pollution and dental caries

Dental caries is a biofilm-mediated, sugar-driven, multifactorial, dynamic disease that results in the phasic demineralization and remineralization of dental hard tissues. It remains a pervasive global health issue, with the highest prevalence among all evaluated diseases according to the Global Burden of Disease Study 2015 (1). Its multifactorial etiology encompasses various physical, biological, environmental, and lifestyle factors—for example, cariogenic bacteria, inadequate salivary flow, insufficient exposure to fluoride, poor oral hygiene, and the crucial event in the clinical course is the initial acquisition of *Streptococcus mutans*.

Notably, environmental factors including SHS, PM and heavy metals have been proved to be implicated in elevating the risk of dental caries, a relationship extensively studied in recent decades. The most important environmental medium of these pollutants is air, followed by other media such as water and soil. PM and SHS are mixtures of numerous chemicals often suspended in the air, ultimately causing contamination of air. In addition, they can settle on surfaces of objects, floor and soil due to gravity, which can lead to contamination of land. When PM2.5 enters water bodies through precipitation, it changes the physical, chemical, and biological properties of the water. Heavy metals derived from automobile exhaust, industrial wastewater, pesticides and other ways are widely present in the air, water and soil, which cause pervasive environmental pollution. Current research on the association between caries and environmental pollution involves limited types of pollutants. In the future, more research can focus on other new pollutants, such as microplastics, volatile organic compounds, ozone, etc.

### 2.1 Secondhand smoke

Tobacco smoking stands as a significant global public health concern, impacting the risk profile for a multitude of health conditions. Over 1 billion people in the world smoke tobacco, and an enormous increase has been predicted, to 1.9 billion in 2025 (18). Recent years have witnessed the establishment of a definitive link between tobacco smoking and the etiology of oral diseases such as dental caries and periodontal disease. Moreover, emerging researches highlight the impact of SHS, a form of indoor air pollution, on the occurrence and progression of various oral diseases. SHS, commonly known as passive smoking, occurs when individuals inhale environmental tobacco smoke (ETS). Alarmingly, nearly half of the world’s children are exposed to passive smoking within their family homes, a factor intricately linked to heightened dental caries risk.

**Figure 1:**
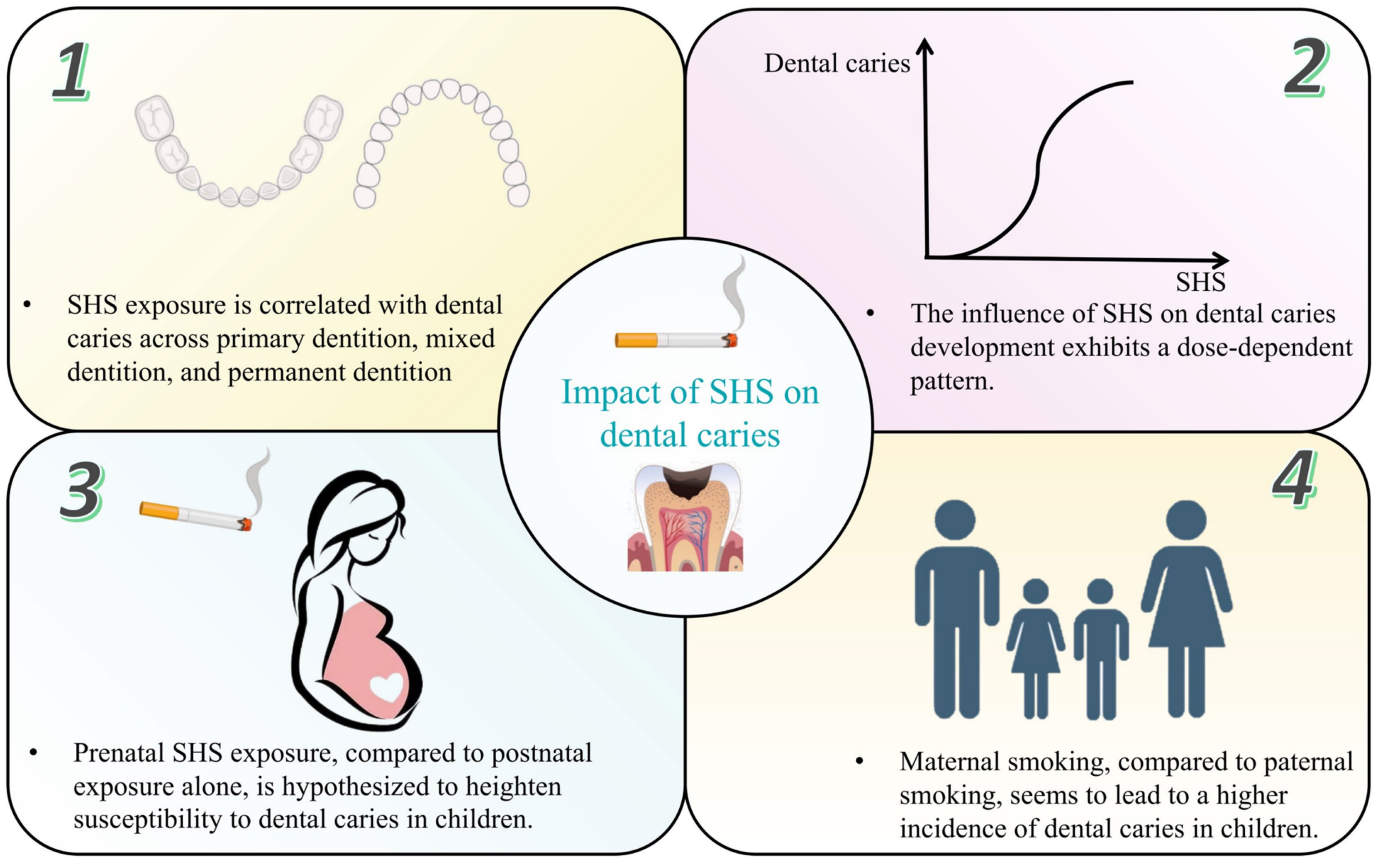
The impact of secondhand smoke on dental caries prevalence. SHS, secondhand smoke.

Numerous epidemiological studies, encompassing case–control, cross-sectional and cohort designs, have corroborated the association between SHS exposure and increased dental caries risk (Table 1). Dental caries was reported using different indices in these studies. The decayed, missing, and filled teeth or surfaces (dmft/dmfc for deciduous dentition, DMFT/DMFC for permanent dentition) indices were the most common, with a score of one or higher signifying dental caries. In addition, the International Caries Detection and Assessment System (ICDAS), early children caries (ECC) and severe early children caries (SECC) were also considered indicators of caries. Cotinine, the primary metabolite of nicotine, is a reliable biomarker to quantitatively measure exposure to ETS and passive smoking. Cotinine will only be present in the body when exposed to tobacco smoke and it can be measured in blood, urine, or saliva. A positive association between salivary/urine/serum cotinine levels and passive smoking, as well as a dose–response relationship, was reported by studies (19–21). In this review, through an extensive literature search, we consolidate the impact of SHS on dental caries prevalence as follows (Figure 1):

1 SHS exposure is significantly correlated with dental caries across primary dentition, mixed dentition, and permanent dentition.

**Table 1:** Studies accessing exposure to secondhand smoke and dental caries risk.

Author Year Country	Source of SHS	Age of participants	Sample size	Study design	Results OR/95% CI/*p* value
Arafa (20) 2023 Saudi Arabia	Household	5–7 years	210	Case–control study	The household smoking group presented with statistically significant higher score of ICDAS (*p* < 0.000).
Misrabi et al. (188) 2023 Syria	Household	10–13 years	284	Cross-sectional study	The number of smokers at home was significantly associated with the DMFT score, dental plaque accumulation, and gingival inflammation (*p* < 0.1).
Mahabee-Gittens et al. (189) 2022 USA	Household	1–11 years	32,214	Cross-sectional study	Children with home SHS and THS exposure were at increased odds of having carious teeth (AOR = 1.74, 95% CI = 1.14–2.65, *p* = 0.010).
Dearing et al. (190) 2022 USA	Prenatal postnatal	4–11 years	1,733	Cross-sectional study	This study does not provide evidence that prenatal or post birth SHS leads to dental caries in children.
Akinkugbe (32) 2021 England	Prenatal maternal	2.5-5 years	1,429	Prospective cohort study	The AOR (95% CI) for offspring caries at for smoking in the first, second and third trimesters were, respectively, 1.16 (0.93, 1.43), 1.11 (0.75, 1.65) and 1.60 (1.09, 2.32).
Saho et al. (191) 2020 Japan	Household	18–19 years	1,905	Cross-sectional study	The risk of DMFT ≥1 was significantly associated with household exposure to SHS ≥ 10 years (OR = 1.50, 95% CI = 1.20–1.87, *p* < 0.001).
Lee et al. (23) 2020 Malaysian	Household	3–6 years	396	Cross-sectional study	Exposed to SHS (AOR = 1.67, 95% CI = 0.168–0.857, *p* = 0.004) were significantly associated with dental caries.
Tang et al. (192) 2020 China	Postnatal household	3 years	283	Cross-sectional study	There might be a potential negative effect of SHS on the deciduous caries (*p* < 0.05).
Nakayama et al. (22) 2019 Japan	Household	3 years	2,277	Cross-sectional study	Exposure to SHS was significantly associated with higher odds of ECC (OR = 1.74, 95% CI = 1.38–2.20, *p* < 0.001) and S-ECC (OR = 1.81, 95% CI = 1.25–2.62, *p* = 0.002).
Goto et al. (27) 2019 Japan	Household	3–6 years	405	Cross-sectional study	Exposure to SHS may have a dose-dependent influence on the development of caries (> 3 pack-years of exposure: OR = 5.55, 95% CI = 2.17–14.22; ≤3 pack-years of exposure: OR = 1.47, 95% CI = 0.56–3.84).
Julihn et al. (193) 2018 Sweden	Prenatal maternal	3 years	73,658	Cross-sectional study	Maternal smoking during pregnancy were predictors of dental caries in preschool children (OR = 1.58, 95% CI = 1.39–1.79).
NN et al. (194) 2017 New Zealand	Prenatal postnatal	15–69 months	44	Cross-sectional study	Participants exposed to ETS were more likely to have dental caries (*p* < 0.05).
Bernabe et al. (195) 2017 Scotland	Postnatal maternal	1–4 years	1,102	Cohort study	The predicted mean difference in dmfs was 1.66 (95% CI = 0.57–2.75) between children of smoking and non-smoking mothers.
Tanaka et al. (196) 2015 Japan	Prenatal postnatal	0–3 years	76,920	Cohort study	The propensity score adjusted hazard ratio between maternal smoking during pregnancy and having no smoker in the family was 1.10 (95% CI = 0.97–1.25).
Tanaka et al. (28) 2015 Japan	Prenatal postnatal	3 years	6,412	Cross-sectional study	Maternal smoking during pregnancy and postnatal SHS exposure may be associated with dental caries in primary dentition (AOR = 1.62, 95% CI = 1.23–2.11).
Nakayama et al. (34) 2015 Japan	Maternal	3 years	1,801	Cross-sectional study	Maternal smoking was significantly associated with the risk of ECC (OR = 1.91, 95% CI = 1.43–2.54).
Majorana et al. (197) 2014 Italian	Prenatal postnatal	24–30 months	2,395	Cross-sectional study	Environmental exposure to smoke during the first year of life was also significantly associated with caries severity (OR = 7.14, 95% CI = 6.07–7.28).
Plonka et al. (198) 2013 Australia	Household	0–36 months	1,017	Longitudinal case–control	Children who showed caries by 30 months were more likely to be exposure to SHS (*p* = 0.02).
Tanaka et al. (25) 2010 Japan	Household	6–15 years	20,703	Cross-sectional study	SHS was independently associated with an increased prevalence of DFT [AOR (95% CI) for former and current light and heavy household smoking were 1.03 (1.00–1.05), 1.04 (1.02–1.05), and 1.04 (1.03–1.06), respectively].
Tanaka et al. (29) 2009 Japan	Prenatal postnatal	3 years	2,015	Cross-sectional study	Both prenatal and postnatal exposure to ETS was associated with dental caries in young children [AOR (95% CI) were 1.43 (1.07–1.91) and 1.25 (1.04–1.50)].
Hanioka et al. (35) 2008 Japan	Parental	3 years	711	Cross-sectional study	The association of ECC with paternal smoking was weaker than with maternal smoking [AOR (95% CI) were 1.52 (1.10–2.30) and 2.25 (1.51–3.37)].
Leroy et al. (199) 2008 Belgium	Parental	3–5 years	2,533	Cross-sectional study	The significant relationship between parental smoking behavior and caries experience persisted after adjusting for the other evaluated variables (OR = 3.36, 95% CI = 1.49–7.58).
Shenkin et al. (200) 2004 USA	Household	4–7 years	637	Cohort study	ETS was associated with an increased risk of caries among children (OR = 3.38, 95% CI = 1.68–6.79, *p* = 0.001).
Aligne et al. (21) 2003 USA	Household	4–11 years	3,531	Cross-sectional study	For dental caries in deciduous teeth, the AOR was 1.8 (95% CI = 1.2–2.7) for the risk of decayed surfaces and 1.4 (95% CI = 1.1–2.0) for filled surfaces.
Williams et al. (33) 2000 England	Parental	3–4.5 years	749	Cross-sectional study	Maternal smoking is a significant factor to be considered as an additional caries risk indicator (OR = 1.54, 95% CI = 1.07–2.21).

OR, odds ratio; 95% CI, 95% confidence interval; AOR, adjusted odds ratio; RR, risk ratio; SHS, secondhand smoke; THS, thirdhand smoke; ICDAS, International Caries Detection and Assessment System; DMFT score, the number of decayed, missing or filled permanent tooth due to dental caries; dmfs, the number of decayed, missing or filled primary tooth surface due to dental caries; ECC, early childhood caries; S-ECC, severe early childhood caries; ETS, environmental tobacco smoke.

Most of the participants in the studies were infants and preschoolers aged 0 to 6 years, which is considered more susceptible to caries, with immature immune systems, lower salivary flow, thinner enamel and potentially extended durations of passive smoking at home. Exposure to SHS was significantly associated with higher odds of ECC and SECC (22), with statistically significant higher score of ICDAS (20) and dmft (23). A systematic review of 25 pertinent studies, culminating published in 2023, strongly underscores a substantial link between dental caries in deciduous dentition and SHS (24). For schoolchildren with mixed or permanent dentition, SHS was also proved to be independently associated with an increased prevalence of caries. According to a cross-sectional study including 20,703 schoolchildren aged 6–15 years in Japan, household smoking exposure was associated with an increased prevalence of caries not only in those with deciduous but also those with permanent dentition (25). A systematic review and meta-analysis published in 2018 further concluded that an association does exist between passive smoking and a greater presence of caries in children, both in primary and in permanent dentition (26).

2 The influence of SHS on caries development exhibits a dose-dependent pattern, contingent upon factors such as the number of smokers in the household, cigarette consumption at home and smoking frequency.

Goto et al. (27) examined the association of exposure to ETS with dental caries among preschool children. Exposure to ETS was assessed in terms of urinary cotinine concentrations and pack-years of exposure to smoking by parents and other family members at home. The results observed significant dose relationships of the pack-years of exposure to SHS with the presence of dental caries (≤3 pack-years of exposure: OR = 1.47, 95% CI = 0.56–3.84; >3 pack-years of exposure: OR = 5.55, 95% CI = 2.17–14.22). In addition, the study also found that the urinary cotinine level was significantly associated with the presence of dental caries, and the dose–response relationship was significantly positive. Other studies have also assessed the pack-years or pack-months of smoking by family members with regard to dental caries (25, 28, 29). All studies observed a significant dose–response relationship similar to Goto’s study.

3 Prenatal SHS exposure, compared to postnatal exposure alone, is hypothesized to heighten susceptibility to dental caries, particularly ECC and SECC.

In 2021, Zhong et al. (30) conducted a systematic review and meta-analysis on this topic. They concluded that there was a significant correlation between maternal smoking during pregnancy and childhood caries both in cross-sectional studies (OR = 1.57, 95% CI = 1.47–1.67) and longitudinal studies (OR = 1.26, 95% CI = 1.07–1.48). However, they reported more prospective and extensive studies on this theme are needed for verification. In 2024, a latest systematic review and meta-analysis included 2 new cohort studies and 1 new cross-sectional study to have more valid results (31). This systematic review and meta-analysis of 85,159 showed smoking during pregnancy increases the risk of tooth caries in children by 78% (OR = 1.78, 95%CI = 1.55–2.05). Notably, in a 2015 cross-sectional study, Tanaka et al. (28) indicated that maternal smoking during the first trimester of pregnancy poses the most significant risk of dental caries in children. Contradicting the results of Tanaka’s study, in a 2021 cohort study Akinkugbe et al. (32) reported the third trimester of pregnancy as the most associated trimester with caries in children.

4 Maternal smoking, in contrast to paternal smoking, appears to steer children toward an escalating trajectory of dental caries incidence attributable to household smoking exposure.

The mother may influence her child more strongly than other family members, as the mother is likely to spend a longer time with the child. Additionally, a transmission of *S. mutans* from smoking mothers to their children may be considered an impact of maternal smoking on pediatric dental caries. It is concluded that maternal smoking is considered as an additional risk indicator beyond social class when predicting caries risk in young children (33). According to a cross-sectional study including 1,801 3-year-old Japanese children, maternal smoking was significantly associated with a higher prevalence of dental caries compared to paternal smoking (paternal smoking OR = 1.40, 95% CI = 1.12–1.75, maternal smoking OR = 2.52, 95% CI = 1.98–3.22) (34). Similarly, another study conducted in Japan reported that dental caries prevalence in 3-year-old children was associated with the smoking status of parents. The association was weaker with respect to paternal than maternal smoking (paternal smoking OR = 1.52, 95% CI = 1.01–2.30, maternal smoking OR = 2.25, 95% CI = 1.51–3.37), but the impact of smoking of other members of the household cannot be excluded (35).

The mechanisms underlying the adverse effects of SHS on dental caries remain elusive. Mosharrafian et al. (36) suggested that passive smoking contributes to increased dental plaque accumulation, akin to active smoking mechanisms. Nicotine present in tobacco fosters the growth and adhesion of *S. mutans* directly to tooth surfaces, thereby elevating the prevalence of this cariogenic bacterium among passive smokers (37, 38). Strong evidence also indicates a heightened transmission of *S. mutans* from smoking parents, particularly mothers, to infants during infancy (39, 40). Moreover, nicotine exposure has been linked to enhanced biofilm formation, increased extracellular poly-accharide precipitation, reduced vitamin C absorption, and heightened acidogenic bacterial growth (41). Passive smoking is further associated with an increased risk of recurrent upper respiratory tract infections and mouth breathing, leading to xerostomia characterized by reduced salivary flow rate and diminished saliva protective factors (42, 43). In the embryonic tooth development phase, nicotine has been reported to impede mineralization of human dental pulp cells under prenatal ETS exposure, thereby heightening susceptibility to dental caries (44). Another contributing factor is the association of smoking habits with poor dietary choices, unhealthy lifestyles, and low health awareness (45). Consequently, schoolchildren exposed to passive smoking may emulate their parents’ behavior, impacting their oral health outcomes (25).

### 2.2 Particulate matter

PM is a complex mixture of solid, liquid or solid–liquid particles suspended in the air, which “affects more people than any other pollutant” as WHO state. PM is primarily derived from the combustion processes involving coal, gasoline, and diesel fuels in vehicular and industrial contexts. It is generally categorized by its mean aerodynamic diameter as PM10 (PM <10 μm in diameter), PM2.5 (<2.5 μm) or ultrafine particles (<0.1 μm). High levels of PM in the air drastically increased the number of people with cardiovascular diseases (46) and chronic obstructive pulmonary disease (47). Moradi et al. (48) aimed to estimate the health effects attributed to PM2.5 pollutants in the air of Ardabil in 2018 (using Air Q + model). The results suggested that PM2.5 exposure even at low concentrations was associated with an increased risk of overall mortality and specific cause mortality and hospital admissions for respiratory and cardiovascular diseases. Otherwise, PAHs as part of PM can cause many complications in humans, including respirations (49), cardiovascular diseases (50) and increased risk of cancer (51).

Oral and nasal cavity are the first channel for PM to enter human body during breathing. In addition, data has indicated that PM2.5 can also deposit in the oral cavity apart from lungs (52). This finding suggests that oral cavity is likely to be one of the fragile organs while exposed to PM especially PM2.5. Additionally, previous studies support that PM can induce reactive oxygen species (ROS) and oxidative stress which contributes to many biological events such as cellular dysfunction, inflammation and oxidative damage (53). During recent years, oxidative stress is reported to involve in the development of many oral diseases such as dental caries, lichen planus, oral cancer, and most importantly chronic periodontitis (54, 55). Chronic oxidative stress and ROS in oral cavity as well as acidic pH on dental enamel surface due to the metabolic activities of bacterial plaque are the major contributors in the development and progression of dental caries (56). The salivary antioxidant capacity was found significantly lower in children with dental caries compared to their caries free counterpart (57). Thus, it’s a promising clue that PM induce caries through ROS-mediated oxidative stress. What’s more, PM including PM2.5 and PM10 have been demonstrated to absorb and condense high concentrations of heavy metals such as lead and cadmium which are associated with increased risk of dental caries as described below (58, 59). A study reported the contents and characteristics of atmospheric PM2.5-bound heavy metals in the northern part of the Persian Gulf from December 2016 to September 2017 (58). The average concentration levels of seven measured metals (Cd, Co, Cr, Fe, Ni, Pb, and V) in the PM2.5 samples were in the range of 6.03 ng/m^3^ to 1,335.94 ng/m^3^, and the order of their concentration was Fe > Ni > Pb > Cr > Cd > V > Co. In addition, it’s well-known that a persistent acidic state in the mouth can lead to tooth demineralization and increase the risk of dental caries. At the same time, several studies have reported the strong acidity of PM2.5, so it is reasonable to assume that long-term exposure to PM2.5 may lead to oral pH disturbances, which can lead to the occurrence of dental caries (60).

In conclusion, these findings provide preliminary evidence in support of the views of underlying interlinkage between PM2.5 and oral health status. Although existing evidence remains not compelling, the potential of PM2.5 to induce damages in oral health should not be overlooked. Future research such as cross-sectional study and prospective cohort study should be designed to examine this plausible hypothesis. If the relationship between PM2.5 exposure and caries is confirmed, and the underlying mechanism is demonstrated, it will provide an important basis for controlling environmental PM2.5 as a public intervention to reduce the incidence of dental caries in the community.

### 2.3 Heavy metals and other chemical pollutants

Chemicals including heavy metals, organochlorine and organophosphate pesticides, organic solvents, pesticide et al. are widespread in the global environment and are proved to cause adverse effects to human health. Chemical pollutants are responsible for 1.8 million deaths annually worldwide, a figure likely underestimated due to their pervasive presence in the modern environment, according to the Global Burden of Disease Study 2019 (5). Among them, heavy metals are reported to be highly toxic, widespread and persistent in our environments. They also have numerous exposure routes, including food ingestion like wheat (61), rice (62), even sugar (63), urban dust inhalation (64), and dermal absorption (65), subsequently inducing some health effects. There have been increasing environmental and public health concerns resulting from heavy metals contamination in recent times. Using data collected in the 2017–2020 National Health and Nutrition Examination Survey (NHANES), Akinkugbe et al. (66) examined the association between environmental metals and dental caries among U.S. children and adolescents. The results showed that increased exposure to a metal mixture was significantly related to an increased prevalence of dental caries.

Among the many metal mixtures, lead is one of the most toxic and pervasive pollutants in society and mainly accumulated in bones and teeth in humans. Even maternal exposure to lead decades before pregnancy can subsequently result in lead exposure of the developing fetus due to its long half-life of approximately 62 years. Using data collected in the NHANES III including 24,901 participants, Moss et al. (67) suggested that the blood lead level was significantly associated with the number of decayed teeth for both deciduous and permanent teeth in all age U.S. groups. In 2000s, two cross-sectional studies consistently supported the association between lead exposure and caries prevalence and pointed that this association was stronger in primary dentition than in permanent dentition (68, 69). The correlation between blood lead levels and caries in deciduous teeth was confirmed in later studies (70), while that in permanent teeth was not statistically significantly (71, 72). In terms of mechanism exploration, Hou et al. (73) reported the chronic lead exposure can reduce salivary sialic acid level, attenuate oral anti-inflammatory potential and increase the risk of dental caries among preschool children.

In addition to lead exposure, cadmium is also a widespread environmental contaminant. Cadmium enters the body mainly via respiration and the digestive tract and even low-dose cadmium exposure can be potentially toxic after long-term accumulation due to the long half-life of approximately 10–30 years. Arora et al. (74) conducted related epidemiological experiments using data from the NHANES III in America and proposed that environmental cadmium exposure may be associated with increased risk of dental caries in deciduous teeth. In conclusion, above findings may help in part to explain the comparatively high levels of dental caries observed in the cities where exposure to lead or cadmium is common.

Apart from heavy metals, the negative impact of radiation exposure on the general health of children has frequently been described, but only little scientific insight has been gathered on the development of oral diseases. A systematic review published in 2019 analyzed the data concerning the caries prevalence in children born and permanently residing in Chernobyl fallout areas. The results showed that although individual studies demonstrated a greater prevalence of dental caries in children residing in radiation-contaminated areas, no conclusive statement is possible regarding the effect of small dose radiation on the dentition (75). Hence, further high-quality epidemiologic investigations are needed.

## 3 Environmental pollution and periodontal diseases

Periodontitis, characterized by inflammatory disorders affecting the supporting tissues surrounding the teeth, significantly impacts tooth loss and affects a substantial portion of the population. Peri-implantitis is an inflammatory damage that occurs in the soft and hard tissues around the implant which can eventually lead to the loosening and falling of the implant. Recent research has illuminated the relationship between environmental pollution and health outcomes, significantly advancing our understanding of the association between periodontal diseases and environmental factors.

### 3.1 Secondhand smoke

Given smoking’s recognized role as a major preventable risk factor in periodontal disease, researchers have explored whether SHS also contributes to the prevalence of periodontitis. In 2001, Arbes et al. (76) conducted a cross-sectional study about the relationship between SHS and periodontitis for the first time. This result suggested that passive smoking may also have a harmful effect on periodontal health. Subsequent studies further confirmed this relationship (Table 2). For these studies, the effect of SHS on periodontitis can be summarized as follows:

1. SHS can influence the gingival index (GI), clinical attachment level (CAL), probing depth (PD) and tooth loss.
2. The effect of SHS for periodontitis is dose-dependent.

**Table 2:** Studies accessing exposure to secondhand smoke and periodontitis risk.

Author Year Country	Age of participants	Sample size	Study design	Results OR/95% CI/*p* value
Umemori et al. (201) 2020 Japan	≥65 years	18,865	Cross-sectional study	The OR for having no teeth rather than having ≥20 teeth among the participants with daily exposure to SHS was 1.35 (95% CI = 1.08–1.68, *p* < 0.01).
Karsiyaka Hendek et al. (83) 2019 Turkey	6–12 years	180	Cross-sectional study	The PI and GI values were significantly higher in SHS-exposed children (*p* = 0.000).
Sutton et al. (202) 2017 USA	≥30 years	4,329	Cross-sectional study	There was a 28% increase in the odds of periodontitis for those with any ETS exposure (*p* = 0.01, 95% CI = 1.06–1.55).
Sanders et al. (203) 2013 USA	Adults	3,137	Cross-sectional study	Odds of periodontitis for those exposed to SHS were elevated 2-fold relative to those who were unexposed (OR = 2.03, 95% CI = 1.30–3.20).
Sutton et al. (204) 2012 USA	Adults	3,137	Cross-sectional study	Adults with high ETS exposure (cotinine ≥1.5 ng/mL) had more than twice the odds of periodontitis (OR = 2.3, 95% CI = 1.3–4.1).
Erdemir et al. (82) 2010 Turkey	6–12 years	109	Cross-sectional study	The mean CAL was significantly less in SHS-exposed children (0.09 mm; *p* < 0.05).
Nishida et al. (205) 2008 Japan	Adults	200	Cohort study	Insignificantly higher periodontitis OR in involuntary smokers (OR = 2.23, 95% CI = 1.03–4.83) relative to non-smokers.
Nishida et al. (206) 2006 Japan	Adults	273	Cross-sectional study	Passive smoke exposure leads to elevation of salivary markers related to periodontitis including IL-1beta, albumin and AST levels.
Yamamoto et al. (207) 2005 Japan	Adults	273	Cross-sectional study	Significantly higher periodontitis OR in passive smokers relative to non-smokers (OR = 2.87, 95% CI = 1.05–7.82).
Tanaka et al. (208) 2005 Japan	Pregnant women	1,002	Cross-sectional study	Passive smoking at home was associated with an increased prevalence of tooth loss in Japanese young adult women (AOR = 1.79, 95% CI = 1.08–2.94).
Arbes et al. (76) 2001 USA	≥18 years	6,611	Cross-sectional study	The AOR of having periodontal disease were 1.6 (95% CI = 1.1–2.2) times greater for persons exposed to ETS.

OR, odds ratio; AOR, adjusted odds ratio; 95%CI, 95% confidence interval; SHS, secondhand smoke; ETS, environmental tobacco smoke; PI, periodontal index; GI, gingival index; CAL, clinical attachment loss; IL-1beta, interleukin-1beta; AST, aspartate aminotransferase.

However, the correlation between SHS and periodontitis is still under debate (77–79). A recent systematic review including both epidemiological and *in vitro* studies by Javed et al. (80) stated that the association was inconclusive and called for additional studies. For adults, Akinkugbe et al. (81) conducted systematic review and meta-analysis to summarize the epidemiological evidence on SHS exposure and prevalent periodontitis endpoints among nonsmokers. The results pointed out that although the findings are consistent with a positive association, there was no meaningful difference in summary estimate for studies reporting CAL and/or PD endpoint (*n* = 6; random effects POR = 1.34 [0.93, 1.94]) as opposed to tooth loss (*n* = 2; random effects POR = 1.33 [0.52, 3.40]). After analyzed, this review considered that the magnitude of association depended mostly on the method of ETS assessment (cotinine-measured exposure stronger than self-reported exposure).

On the other hand, for children and adolescents, study findings regarding the impact of SHS on periodontal diseases are also controversial, even in studies conducted in the same country (82, 83). A systematic review and meta-analysis published in 2022 revealed that children and adolescents exposed to SHS exhibited significantly higher levels of GI compared to unexposed (SMD = 1.03, 95% CI = 0.17–1.89), but no difference was observed for PD (SMD = 0.34, 95% CI = 0.14–0.82), with overall very low certainty on evidence (84). The meta-analysis also reported that there is a necessity for further research to draw conclusions on other pertinent periodontal parameters such as bleeding on probing and CAL. In conclusion, the association between ETS and periodontal disease remains debatable and requires further investigations.

Although scientists are also interested in the effects of SHS on peri-implantitis, current studies are still limited to animal experiments. In 2002, trials conducted by Nociti et al. (85, 86) revealed that intermittent cigarette smoke inhalation may lead to poor bone quality around titanium implants particularly in the cancellous bone area. Moreover, experiments conducted by César-Neto et al. (87) showed that nicotine and cotinine are the main molecules which lead to negative effects. In addition, another study proved that cigarette smoke inhalation amplified the deleterious effects of estrogen deficiency on bone healing around titanium implants (88). To find the possible mitigation methods, scholars designed relevant experiments. Correa et al. (89) did aluminum oxide blast surface treatment and found that it can increase the degree of bone-to-implant contact but cannot overcome the detrimental effect of tobacco smoke on bone around titanium implants. A systematic review and meta-analysis of nine preclinical studies published in 2018 assessed the influence of involuntary cigarette smoke inhalation on osseointegration of dental plants (90). The results showed that significant differences could be observed for bone-to-implant contact for test subjects in cancellous (*Z* = −4.08, *p* < 0.001) and cortical bone (*Z* = −4.31, *p* < 0.001) respectively.

In recent years, the use of electronic cigarette (e-cigarette) has increased particularly among adolescents. E-cigarettes are electronic nicotine delivery systems which mimic tobacco smoking without the combustion of tobacco. While the oral health sequelae of conventional smoking are well-established, information on the effects of e-cigarette smoke on oral health is still scarce. Over the past few years, there have been an increasing number of studies conducted to explore oral health effects of e-cigarette, while epidemiological studies highlight concerns over oral dryness, gingival and periodontal diseases (91, 92). Although the hazardous substances released by e-cigarettes are lower than traditional cigarettes, secondhand exposure to e-cigarette aerosols is not completely benign for bystanders. According to a cross-sectional study on a representative sample of the population aged ≥15 years in 12 European countries, 16.0% of e-cigarette non-users were exposed to secondhand e-cigarette aerosol in any indoor setting at least weekly (93). Two studies revealed that the levels of airborne nicotine and cotinine concentrations in the homes with e-cigarette users were higher than control homes (94, 95). The results showed that non-smokers passively exposed to e-cigarette absorbed nicotine. Additionally, the exposure to primary and secondhand e-cigarette aerosol is proved to be related to higher metal concentrations in the biological samples (96) which may cause potential damage to oral health. In conclusion, the adverse effects on oral health of long-term primary and secondhand exposure to e-cigarette are yet to be determined. Additional clinical and animal-exposure model research is critically needed to validate this association as the use of e-cigarette continues to grow.

**Figure 2:**
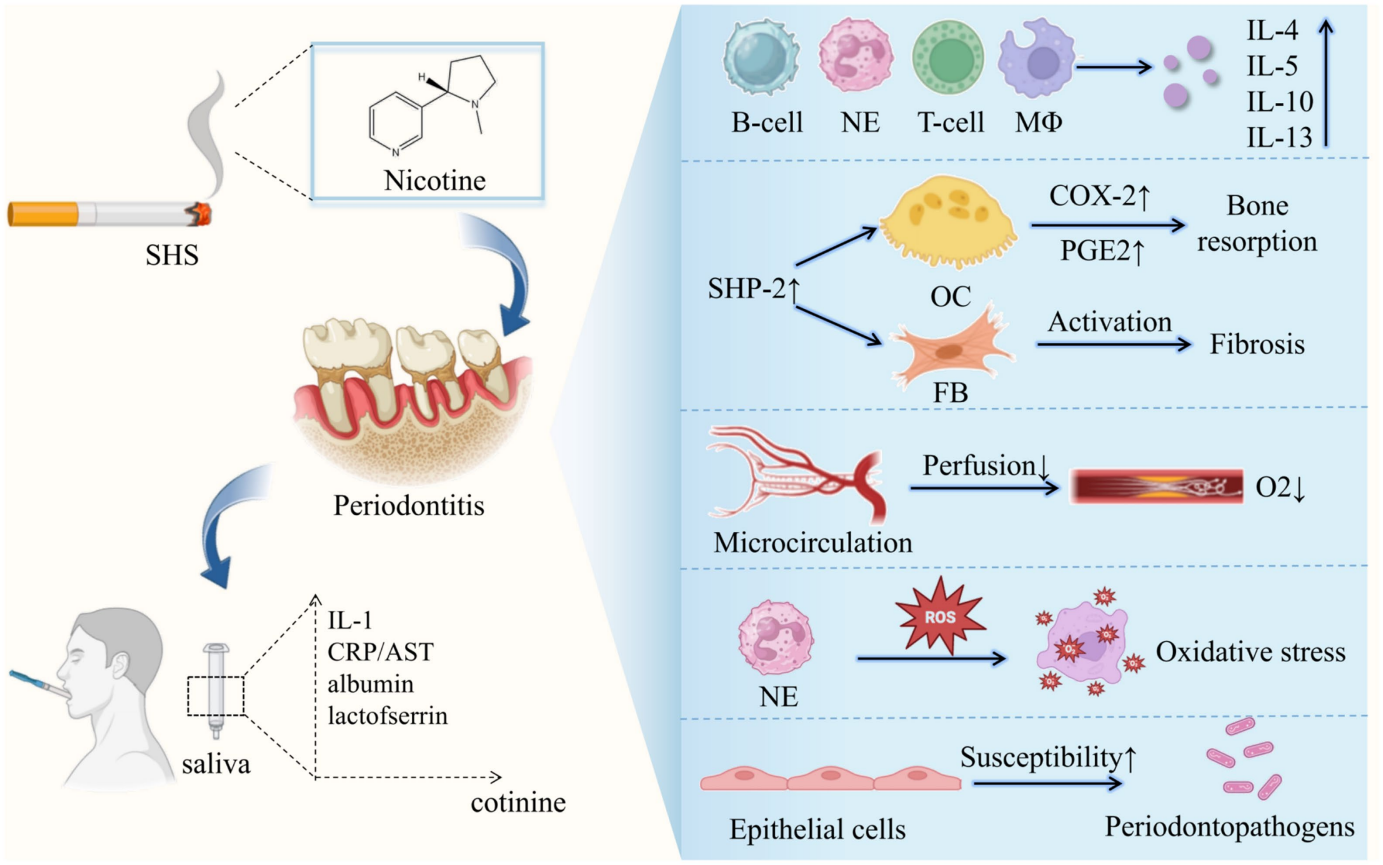
Potential mechanisms linking secondhand smoke and periodontitis. SHS, secondhand smoke; CRP, C-reactive protein; IL-1, interleukin-1; AST, aspartate aminotransferase; NE, Neutrophils; MΦ, macrophage; OC, osteoclast; FB, fibroblast.

Diverse mechanisms are known to interpret the detrimental periodontal effects of passive smoking, mainly including the alteration of both microorganism and host response (Figure 2). Exposure to SHS is proved to suppress the function of gingival fibroblasts and B-cells, impair epithelial cell growth and increase the production of interleukin IL-4, IL-5, IL-10 and IL-13 resulting in increased inflammatory burden and potential alveolar bone loss (80). Numerous compounds in ETS including nicotine, ammonia, nitrogen, and sulfur oxides are demonstrated to have likely harmful effects on periodontal tissues in clinical, vitro and animal experimental studies. Eramo et al. (97) revealed the possible relationship between the salivary concentration of cotinine and the pathogenesis of periodontal disease in passive smokers for the first time. Later, cell experiments conducted by Teughels et al. (98) indicated that the susceptibility of epithelial cells to be colonized by periodontal pathogen could be altered by nicotine, cotinine, or cigarette smoke extract (CSE) in a time-dependent, species-specific manner. What’s more, nicotine, cotinine and CSE can directly induce superoxide generation by otherwise unstimulated neutrophils, stimulating ROS release and oxidative stress mediated tissue damage (99, 100). Sadaoka et al. (101) found that nicotine can increase chromogranin A (ChgA) production of human periodontal ligament-derived fibroblasts, which is involved in the immunomodulation system. What’s more, nicotine can induce a peripheral vasoconstrictive effect that minimizes oxygen delivery to tissues, leading to impairment of periodontal health (102). There are also animal experiments proposing that SHS increased alveolar bone loss in periodontitis with enhanced expression of COX-2 and SHP-2 in periodontal tissues (103).

### 3.2 Particulate matter

In addition to SHS, other types of air pollution such as PM can also contribute to various periodontal health symptoms. C-reactive protein (CRP) is a highly sensitive biomarker of inflammation, which has been widely used to estimate the presence and the intensity of inflammation (104). The association between PM and high-sensitivity C-reactive protein (hs-CRP) has been well documented in epidemiological studies (105). Moreover, recent studies have linked periodontitis to elevated hs-CRP levels compared to periodontally healthy individuals (106). Yang et al. (107) conducted a cross-sectional study to investigate whether PM2.5 exposure had greater effects on increasing hs-CRP among periodontitis patients. The results indicated that personal exposure to PM2.5 significantly increased hs-CRP. Nonetheless, the presence of periodontal disease led to a considerably increased effect magnitude by more than eight folds, suggesting that periodontal patients are probably more susceptible to PM2.5-induced hs-CRP release.

Furthermore, evidence has demonstrated that the carbon components of PM2.5, including elementary carbon and organic carbon, can lead to the overproduction of oral ROS (108, 109). Accumulating evidence shows that periodontitis patients have high level of ROS production and the excessive production of ROS plays a key role in periodontitis-related tissue destruction, among which ROS directly exert cytotoxic and oxidative damage to tissues (110). Therefore, despite lack of direct evidence, it is reasonable to assume that exposure to PM2.5 possibly cause adverse inflammatory reactions in periodontal tissues and promote periodontal tissue damage by increasing ROS concentration within the mouth. A cohort study conducted in 2023 investigated the association among air pollutants, meteorological factors, and periodontal diseases simultaneously (111). The results revealed that PM2.5 exposure increased the risk of periodontitis (RR = 1.049, 95% CI = 1.004–1.096) and older people patients with gingivitis and periodontitis were both vulnerable to PM2.5 exposure. Besides, the adverse effects of air pollutants exposure on periodontal diseases may vary depending on meteorological factors including ambient temperature and humidity.

**Figure 3:**
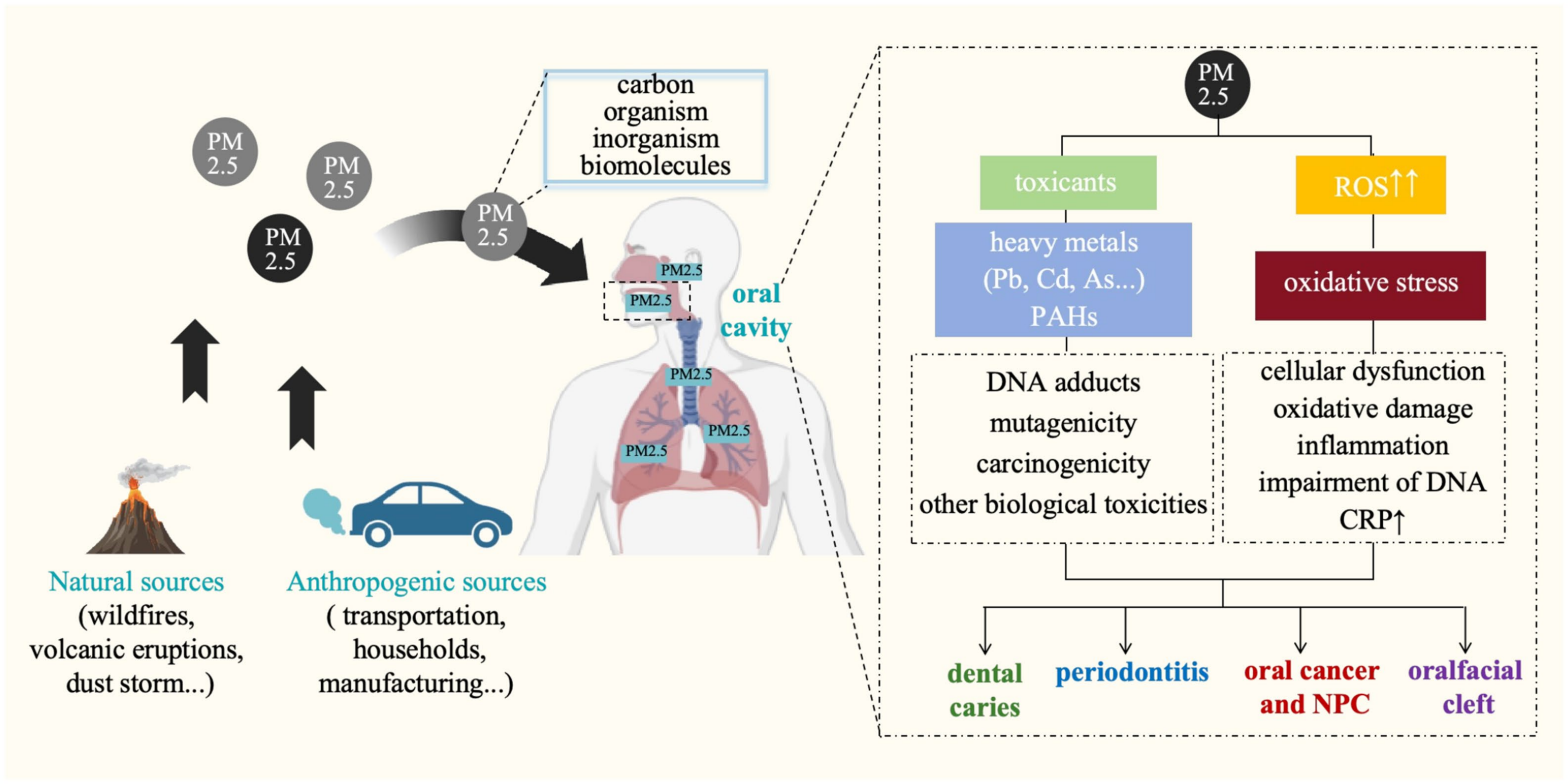
Potential mechanisms linking particulate matter and oral diseases. ROS, reactive oxygen species; Pb, lead; Cd, cadmium; As, arsenic; PAHs, polycyclic aromatic hydrocarbons; CRP, C-reactive protein; NPC, nasopharyngeal cancer.

In conclusion, although the exact mechanism remains unclear, PM2.5 is preliminarily proven capably contributing to adverse inflammatory responses in periodontium, promoting the damage of periodontal tissues (Figure 3). Future studies are essentially needed to examine the causal relationship between exposure to PM2.5 and the development of periodontal disease.

### 3.3 Heavy metals and other chemical pollutants

Heavy metals also have certain effects on periodontal health. Animal experiment demonstrated significant, time-dependent effects of cadmium on periodontal bone resorption (112). Arora et al. (113) analyzed the data from NHANES III in America and examined the relationship between environmental cadmium exposure and periodontal disease in American adults. The results showed that a 3-fold increase in urinary cadmium concentrations was associated with 54% greater odds of prevalent periodontal disease (OR = 1.54, 95% CI = 1.26–1.87). Another two cross-sectional studies conducted in 2020s in America demonstrated that both high lead and cadmium concentrations in serum and urinary have a significant association with periodontitis (114, 115). Additionally, by analyzing data from the NHANES IV in Korea, two studies showed that the ORs for periodontitis were significantly associated with serum cadmium and lead levels (116, 117). In 2023, a study conducted in China investigated the association between heavy metals in tap water and periodontitis in a nationally representative sample (118).

Apart from heavy metals, phthalates are widely used plasticizers, which were identified as risk factors in the development of many human diseases. Wu et al. (119) found that exposure to multiple phthalates was positively associated with periodontitis in US adults between 30 and 80 years old by activating NFκB pathway.

## 4 Environmental pollution and orofacial clefts

OFCs are among the most frequent birth defects with a complex etiology, which can be unilateral or bilateral, complete or incomplete, and may involve the lip only, the palate only, or both. In China, the incidence of OFCs is notably high, which is believed to be linked to environmental pollution and disparities in economic and medical development. Data on perinatal infants and children with cleft lip and/or cleft palate from 2015 to 2018 in Guangdong province of China found that the incidence of OFCs was 7.55/10,000 (120). Growing evidence points to a link between maternal exposure to environmental pollutants during pregnancy and increase risk of OFCs.

### 4.1 Secondhand smoke

Smoking has been extensively linked to various congenital anomalies, including OFCs. Numerous studies have investigated the association between smoking and non-syndromic orofacial clefts (NSOFCs), consistently finding a positive correlation with both maternal active and passive smoking.

Although the magnitude of this association varies across studies, meta-analysis has strengthened the validation of these risks associated with passive smoking. For instance, a systematic review and meta-analysis in 2014 highlighted a 1.5-fold increase in the risk of NSOFC due to maternal passive smoking, similar to the magnitude of risk reported for active smoking (121). Another systematic review and meta-analysis recently published in 2023 updated the literature on the association between SHS and NSOFCs. This meta-analysis findings revealed that maternal SHS exposure was linked to a more than 1.5-fold increase in the risk of having a child with NSOFC, exhibiting a higher odds ratio compared to maternal active smoking (122).

Multiple hypothesized mechanisms explain how tobacco smoke escalates the risk of OFCs. Tobacco smoke contains nicotine and various teratogens like PAHs, dioxins, carbon monoxide, pesticides, and heavy metals such as cadmium. The link between heavy metal exposure and OFCs has been validated in both animal models and human studies. Heavy metals likely act as teratogens by inducing oxidative stress and disrupting redox-sensitive signaling pathways (123, 124). The research on the mechanism of environmental pollution on OFCs has gone deep into the genetic level. By genome wide association studies, scientists find that exposure to SHS can influence the expression of some genes, such as SPRY2 (125), SLC2A9 and WDR1 (126), BMP4 (127), RUNX2 (128), miR-140 (129), SUMO1111 (130), IRF6 (131), MYH9 (132), TGFbeta3 (133), and MSX1 (134).

### 4.2 Particular matter and gaseous air pollutants

Investigating whether PM or other gaseous air pollutants can traverse the placenta to reach the fetus is a crucial inquiry. Bongaerts et al. (135) found that maternally inhaled carbonaceous air pollution particles can cross the placenta and then translocate into human fetal organs during gestation. This phenomenon represents a potential mechanism elucidating the harmful health impacts of air pollution starting from early life. Upon entering fetal organs, PM may trigger adverse effects by instigating oxidative stress and provoking inflammatory responses.

Maternal exposure to ambient air pollution has been proved to be related to oral clefts in offspring, however the epidemiologic evidence is equivocal. In 2023, in order to further analyze the relationship between air pollutants and the occurrence of OFCs, Huang et al. (136) conducted systematic review and meta-analysis including 11 case–control and cohort studies to explore the possible correlation between common environmental air pollution such as PM2.5, PM10, SO_2_, NO_2_, O_3_, CO and the occurrence of neonatal OFCs. Their findings revealed a significant statistical correlation between exposure to PM10, PM2.5, O_3_ and the risk of OFCs in the second month of pregnancy. Conversely, no association was found between exposure to SO_2_, CO, NO_2_ during pregnancy and the risk of OFCs. These results align with the conclusions of Rao et al. (137) previous meta-analysis, indicating that O_3_ exposure increases the risk of OFCs.

### 4.3 Heavy metals and other chemical pollutants

The presence of toxic elements is linked to the development of OFCs. If mothers are exposed to toxic elements during pregnancy due to their milieu or occupations, infants might be at a higher risk for OFCs. Notably, heavy metals including mercury, cadmium, lead, and arsenic, nickel, which are well-known toxic elements, have all been demonstrated to breach the placental barrier. Consequently, the adverse impacts of these elements on reproductive health have been extensively documented.

A case–control study explored the relationship between lead and cadmium concentrations in umbilical cord tissues and the risk of OFCs, revealing that heightened utero exposure to these metals may elevate the risks of OFCs in newborns (124). In blood samples, one cohort study found that exposure to lead and cadmium during pregnancy was not associated with isolated cleft lip and palate at the low exposure level (138); another cross-sectional study indicated a significant association between elevated lead and cadmium levels in infants’ blood and the risk of OFCs (139). Additionally, one case–control study showed that prenatal exposure to cadmium and lead, as reflected by their concentrations in placental tissues, is associated with an increased risk for neonatal OFCs and showed a dose–response pattern (123). Apart from lead and cadmium, the effects on OFCs of other heavy metals including Mercury (123, 138, 139), Barium (140, 141), Strontium (140, 142), Uranium (143, 144), Thorium (144), Arsenic (124, 145) and Nickel (124, 146) have also been investigated, although very little information about the OFCs related to these metals mentioned above is available and the results keep controversial.

Additionally, pesticides are among the exposures reportedly associated with increased risks for OFCs. The risk of OFCs associated with maternal and paternal pesticide exposures separately was examined by conducting a meta-analysis of studies published from 1966 through 2005 (147). The results reported that maternal occupational exposure was associated with an increased risk of OFCs (OR = 1.37, 95% CI = 1.04–1.81), whereas the estimates were somewhat weaker for paternal occupational exposures (OR = 1.16, 95% CI = 0.94–1.44). Recently, more epidemiologic studies had explored the associations between parental occupational pesticide exposure and OFCs. Consistent with the previous studies, maternal occupational pesticide exposures suggest positive associations with OFCs whereas those for paternal exposures are mixed (148–150). Yang et al. (151) firstly explored the association between in utero exposures to organochlorine pesticides and the occurrence of OFCs and the conclusion did not support this association. Studies also evaluated the effects of parental occupational exposure to more chemical contaminants, including organic solvents, dust, gases, and fumes on the development of OFCs (149, 150, 152). Among them, a meta-analysis examined the association between maternal occupational exposure to solvents, pesticides and metals as assessed by expert-based assessment and congenital anomalies in the offspring (152). The results found that there was an association between maternal occupational exposure to solvents and OFCs, but no associations between occupational exposure to pesticides or metals and OFCs in the offspring in this meta-analysis. Larger studies are needed to confirm this finding.

## 5 Environmental pollution and cancer

### 5.1 Secondhand smoke

Cancer remains a leading cause of mortality globally, posing a significant challenge to increasing life expectancy worldwide. Head and neck cancer (HNC) ranks among the most prevalent cancers, with approximately 890,000 new cases and 450,000 deaths reported in 2018 according to Global Cancer Statistics (153). HNC primarily affects regions such as the oral cavity, nasopharynx, oropharynx, hypopharynx, and larynx. The established link between tobacco smoking and HNC is well-documented, with SHS also shown to have a carcinogenic effect in non-smokers.

Data from a large case–control study in America suggested a role for SHS in the etiology of HNC (154). Further investigations, such as a case–control study focusing on an East Asian population, also supported the association between SHS and HNC risk, highlighting a potentially stronger link with oral cavity cancer compared to other HNC subsites (155). Idris et al. (156) prospectively evaluated the role of SHS on recurrence and survival in treated HNC patients. The results showed that SHS significantly increased recurrence and decrease recurrence-free survival, indicating SHS exposure as an independent predictor of recurrence and survival after HNC treatment. However, the underlying mechanisms responsible for these negative patient outcomes remain unclear. To delve into the effects of SHS exposure on HNC treatment, Sadhasivam et al. (157) investigated SHS’s influence on cisplatin efficacy in cancer cells. Their findings demonstrated that SHS exposure can enhance cisplatin resistance by altering the expression of proteins involved in multidrug resistance, thereby increasing cancer cells’ ability to evade cisplatin-induced cell death. These results underscore the importance for clinicians to consider SHS exposure’s potential impact on HNC etiology and treatment outcomes.

A systematic review and meta-analysis by Mariano et al. (158) in 2022 further solidified the link between SHS exposure and oral cancer risk. The results found that compared with non-exposed individuals, the duration of SHS exposure of more than 10 or 15 years increased the risk of oral cancer (OR = 2.07, 95% CI = 1.54–2.79, *p* < 0.00001), supporting a causal association between SHS exposure and oral cancer. Nasopharyngeal cancer (NPC) is one of the most common cancers in southern China. Case–control studies conducted in southern China suggested that there was a strong and statistically significant positive association between NPC risk and exposure to substantial SHS especially as a child or as an adult in women (159). What’s more, Chen et al. (160) evaluated the confounding effects of passive smoking on association between tea and oral cancer in Chinese women, showing that tea consumption reduces the risk of oral cancer in Chinese women, but this effect is modified by the carcinogenic effects of passive smoking.

### 5.2 Particulate matter

Since 2013, outdoor air pollution, particularly the PM component, has been officially classified as a carcinogen by the International Agency for Research on Cancer under the World Health Organization. Previous research has demonstrated that ROS-mediated oxidative stress upon exposure to PM2.5 can activate signaling pathways, modulate transcription factors, and damage DNA, thereby initiating and promoting tumor development, including lung cancer and non-lung cancer (161, 162).

In 2019, Chu et al. (163) directly link PM2.5 exposure to an increased risk of oral cancer. Their analysis included 482,659 Taiwanese men over the age of 40 and revealed that high levels of PM2.5 (>40.37 μg/m^3^) were associated with a 43% higher likelihood of developing oral cancer, including cancers of the lips, tongue, cheeks, mouth floor, and hard and soft palate. A meta-analysis of 14 studies was conducted to find out the possible relationships of exposure to mixed air contaminants and oral cancer (164). The meta-analysis results suggested a statistically significant relationship of oral cancer and PM2.5 exposure (OR = 1.13, 95% CI: 1.06, 1.20). Additionally, high concentrations of both coarse PM (PM2.5–10) and fine PM (PM2.5) were significantly linked to a higher risk of NPC in Taiwanese men (165, 166). Study from Yang et al. (167) further underscored the positive association between ambient air pollutants like SO_2_, PM10, and notably NO_2_, and NPC incidence in China.

Overall, there is a compelling case to view PM as a carcinogenic factor contributing to the initiation of oral cancer and NPC. However, several gaps remain, particularly regarding the kinetics of PM in the oral cavity and the underlying mechanisms of malignancies induced by PM. Therefore, further research is necessary to elucidate these problems.

### 5.3 Heavy metals and other chemical pollutants

Chemical pollution, especially in occupational settings, is another significant factor contributing to HNC, particularly NPC, especially in high-incidence areas. The carcinogenicity of toxic heavy metals has been extensively studied and proven through numerous experiments. Long-term and short-term exposure to heavy metals are significantly associated with various types of cancers in humans (168, 169). Chronic exposure to heavy metals via food, tobacco smoking and occupational exposure has long been acknowledged as a factor that can increase the incidence of HNC, including laryngeal and nasopharyngeal cancer, among exposed populations (170, 171). Khlifi et al. (172) investigated the risk of laryngeal and nasopharyngeal cancer associated with arsenic and cadmium in the Tunisian population and found that HNC patients’ blood levels with arsenic and cadmium were significantly higher than those of controls. Peng et al. (173) suggested that cadmium exposure was associated with NPC in a population with a relatively high prevalence in southeast China. These studies highlight the importance of understanding and mitigating the risks associated with heavy metal exposure in relation to HNC incidence.

Dust generated in wood-related processes has long been recognized as a common occupational risk factor associated with cancer (174). Some studies of the occupational population have reported associations between NPC and occupational exposure to wood dust, but the results remain contentious. To address this, two systematic review and meta-analysis analyze the current epidemiological evidence to examine the association between occupational exposure to wood dust and the risk of NPC. Both studies hint at the contributing effect of wood dust upon NPC (175, 176). Apart from dust, the effects of occupational exposure to other substances on NPC are also discovered. In 2021, Chen et al. (177) did a population-based case–control study, consisting of 2,514 incident NPC cases and 2,586 randomly selected population controls, in southern China. The results showed that occupational exposures to dusts, chemical vapors, exhausts/smokes, or acids/alkalis are associated with an excess risk of NPC. Additional, duration-response trends were observed with increasing duration of occupational exposure. In 2009, a working group of the International Agency for Research on Cancer classified formaldehyde as carcinogenic to humans and concluded that formaldehyde causes NPC (178). However, in recent updated re-analysis of this conclusion, Marsh et al. concluded that the results of the original analysis of NPC-risk are misleading because they are based on inappropriate regression analyses and that their updated re-analysis did not support a persistent association between formaldehyde exposure and NPC risk (179). In addition, studies have evaluated the occupational risk status and incidence of lip and tongue cancer across different occupations in the Nordic countries. The results suggested that certain occupational exposures may be carcinogenic factors in the development of tongue or lip cancer (180, 181).

## 6 Environmental pollution and other oral diseases

Dental fluorosis is a developmental disturbance of dental enamel, caused by successive exposures to high level of fluoride during tooth development. Fluoride is one of among chemicals that has been shown to cause significant effects in people through drinking water (182). While fluoride at low concentrations in drinking water is beneficial for teeth development, excessive exposure (greater than the World Health Organization guideline value of 1.5 mg/L) can lead to adverse effects. A national study used the 2013–2014 and 2015–2016 NHANES data suggested that exposure to higher concentrations of fluoride in water and having higher plasma levels of fluoride were associated with a greater risk of dental fluorosis (183). In 2019, Demelash et al. (184) conducted a systematic review and meta-analysis to assess the fluoride concentration in groundwater and the prevalence of dental fluorosis among residents in Ethiopian Rift Valley. Their findings revealed that fluoride levels in groundwater exceeded the standard value (1.5 mg/L), with a relatively high pooled prevalence of dental fluorosis observed. In addition to drinking water sources, in regions such as Yunnan and Guizhou Province in Southwest China, there is increasing evidence indicating that the primary route of fluoride exposure for residents is through coal-burning roasted foodstuffs, particularly roasted pepper and corn. This exposure has been linked to dental fluorosis among children in these areas (185–187).

Although limited, some research has explored the effects of environmental pollution, including air and chemical pollution, on other oral diseases such as tonsil diseases, enamel hypoplasia, oral mucosal disease, bruxism, mumps, pulpal periapical disease, Sjögren’s syndrome, dental erosion, and Bell’s palsy. Table 3 outlines the relationships between these oral diseases and various types of environmental pollution.

**Table 3:** Relationships between other oral diseases and environmental pollution.

Oral diseases	Air pollution		Chemical pollution	Special environment
	SHS	Particulates		
Tonsil diseases	Y (209–211)	Y (212)	Y (213)	N
Enamel hypoplasia	Y (194)	N	N	Fluoride (214) Fluoride and sulfur dioxide (215)
Oral mucosal disease	Y (194, 216–220)	Y (221)	N	Acidic environment (222)
Bruxism	Y (223–225)	N	N	N
Mumps	N	Y (226–228)	N	N
Pulpal periapical disease	N	Y (229, 230)	N	N
Sjögren’s syndrome	N	Y (231)	N	N
Dental erosion	N	N	N	Acidic environment (232)
Bell’s palsy	N	Y (233)	N	N

“Y” means there are literatures supported; “N” means literature not supported.

## 7 Discussion

Environmental pollution and oral diseases are both worldwide health issues that deserve considerable attention. A vast amount of literatures shows that environmental pollutants, including air and chemical pollutants, are important risk factors for oral diseases. This review highlights epidemiological evidence linking environmental pollution and oral diseases such as dental caries, periodontal diseases, OFCs, HNC, as well as other oral diseases. Among numerous environmental pollutants, SHS, PM and heavy metals especially lead and cadmium are of great concern and have been proven to be significantly associated with different oral diseases. Additional contributors, such as radiation pollutants, electronic cigarette, phthalates, gaseous air pollutants, pesticides, solvents, wood dust, formaldehyde and excessive fluoride were investigated, though evidence for their impacts remains limited and often inconclusive. The review also seeks to elucidate potential mechanisms underlying these impacts. Although we have proposed some possible pathogenic mechanisms, including microorganism, inflammation, oxidative stress, genetic influences, and toxicant exposures from heavy metals and other pollutants, more specific details remain to be elucidated in the future.

Most of the epidemiological evidence linking environmental pollution to oral diseases discussed here are from cross-sectional studies. To establish a causal relationship between exposure to environment pollutants and oral diseases, prospective longitudinal studies on this topic with specific and quantified measurement of environmental exposures are encouraged. It is important to investigate the toxic effects of different components of various pollutants and the kinetics after entering the oral cavity. Another important point to note is how exposure is measured. Research should explore reliable and valid method and novel biomarkers for environmental exposure assessment. And at a wide range of exposure levels, more researches should try to establish the dose–response relationship between a pollutant and oral disease. Additionally, it is also critical to control potential confounding variables such as socioeconomic, educational, and behavioral factors. Subsequent research in this area should be meticulously designed to mitigate the potential influence of confounding factors on the results. In terms of mechanism exploration, most studies focus on their effects on general pathways, such as regional inflammation and oxidative stress and lack of detailed mechanisms of the pathogenesis. More exploration is needed to further explain the specific mechanism. In addition, genetic factors may influence the impacts of environmental pollutants on oral diseases, thus influencing disease susceptibility. Therefore, it is necessary to explore the genetic background of patients and the interaction between environment pollutants in future research.

The significance of this research lies in its contribution to understanding how environmental pollution affects oral health outcomes. By elucidating these complex associations, this article provides insights for public health interventions and policy initiatives aimed at reducing the burden of oral diseases linked to environmental factors. Moreover, it underscores the importance of interdisciplinary collaboration between dental professionals, environmental scientists, and public health experts to address these challenges effectively.

Ultimately, environmental pollution is a global problem whose consequences are not yet adequately and fully known and demand a global response. Thus, it is important to raise awareness among government policy makers and the public about environmental pollution as a threat to public health. A larger focus is needed to control pollution and prevent pollution-related disease, with an emphasis on air pollution and hazardous chemical pollution. International organizations and national governments need to prioritize clinical and health-care research into the effects of pollutants and form science–policy interface to guide practice.

## 8 Conclusion

This review provides an in-depth analysis of the association between environmental pollutants and oral health risks, synthesizing current literature on the impact of pollution on oral diseases. Evidence indicates that pollutants such as SHS, PM, and heavy metals contribute significantly to the development of dental caries, periodontal diseases, OFCs, HNC, and other oral conditions. Looking forward, future research in this field should prioritize longitudinal studies to establish a causal relationship, develop advanced biomarkers for environmental exposure assessment, and design targeted interventions for high-risk populations. Emerging technologies, including genomics and advanced environmental monitoring tools, offer promising avenues for unraveling the mechanisms underlying pollution-induced oral diseases. Ultimately, sustained research efforts and policy implementations are critical to mitigate the adverse effects of environmental pollution on oral health. Such initiatives will not only enhance oral health outcomes but also contribute to broader public health improvements on a global scale.
